# Coccidioidomycosis Chorioretinitis

**DOI:** 10.1177/2324709619881561

**Published:** 2019-10-09

**Authors:** Brian Nordstrom, Namgyal Sherpa, Meagan Marshall, Anuj Chawla, Arash Heidari, Royce Johnson

**Affiliations:** 1 Valley Fever Institute, Kern Medical, Bakersfield, CA, USA; 2 American University of the Caribbean, Cupecoy, Sint Maarten; 3 Retina Institute of California, Bakersfield, CA, USA

**Keywords:** coccidioidomycosis, chorioretinitis, endopthalmitis, choroiditis, retinitis

## Abstract

Coccidioidomycosis is an invasive fungus found primarily in the soil of Southwestern United States, Mexico, and Central America. Primary disease mostly presents as a pulmonary disease although multiple organ systems can be affected through lymphohematogenous dissemination, with ocular seeding extremely rare. When present, the anterior segment structures are most commonly affected. Isolated choroid and/or vitreal disease has been reported infrequently. This is a case of chorioretinitis with vitreal involvement.

## Introduction

Coccidioidomycosis is an invasive fungal strain found primarily in the soil of Arizona, California, Mexico, and Central America. Primary inoculation with *Coccidioides* most commonly occurs through inhalation of aerosolized arthroconidia. Therefore, populations with frequent exposure to contaminated soil such as agricultural workers are particularly at risk for infection. Although the most common clinical presentation is pulmonary coccidioidomycosis, alternate organ systems can be affected in disseminated disease through lymphohematogenous spread. Disseminated coccidioidomycosis occurs in approximately 1% of infected patients. For reasons not fully understood, disseminated disease occurs more commonly in those of Filipino or African descent. Secondary organ systems commonly seeded in disseminated disease include skin, soft tissue, bones, and central nervous system.^1,2^ Ocular involvement have been reported; however, it is extremely rare. When ocular involvement is present, it most commonly affects the conjunctiva and the anterior segment structures. Cases of iridocyclitis have been reported. Isolated choroid and/or vitreal disease are even less common. To our knowledge, only 6 cases of coccidioidal chorioretinitis have been reported in patients with disseminated disease.^3,4^ The majority of these cases were reported without vitreal involvement or arose status post vitrectomy.^5^ Our case is a rare presentation of chorioretinitis with vitreal involvement.

## Case Presentation

**Figure 1.**
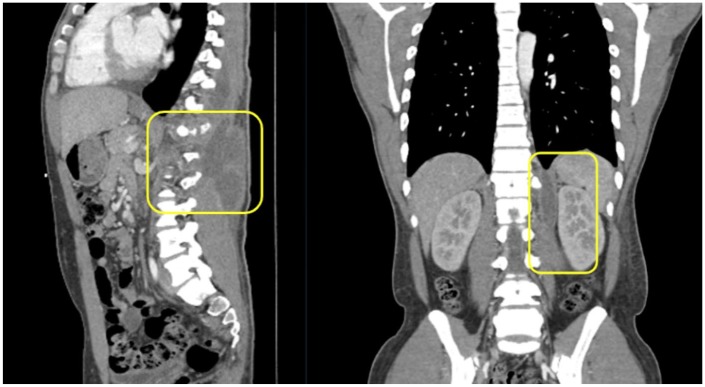
Thoraco-abdominal computed tomography scan showing T7-T12 paraspinal abscess.

**Figure 2.**
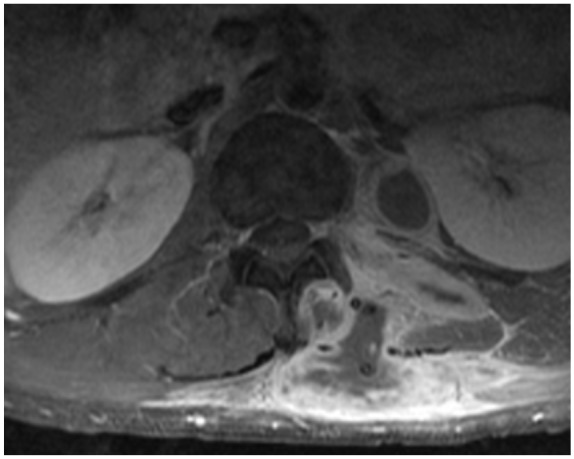
Thoracoabdominal computed tomography scan showing T7-T12 paraspinal abscess.

**Figure 3.**
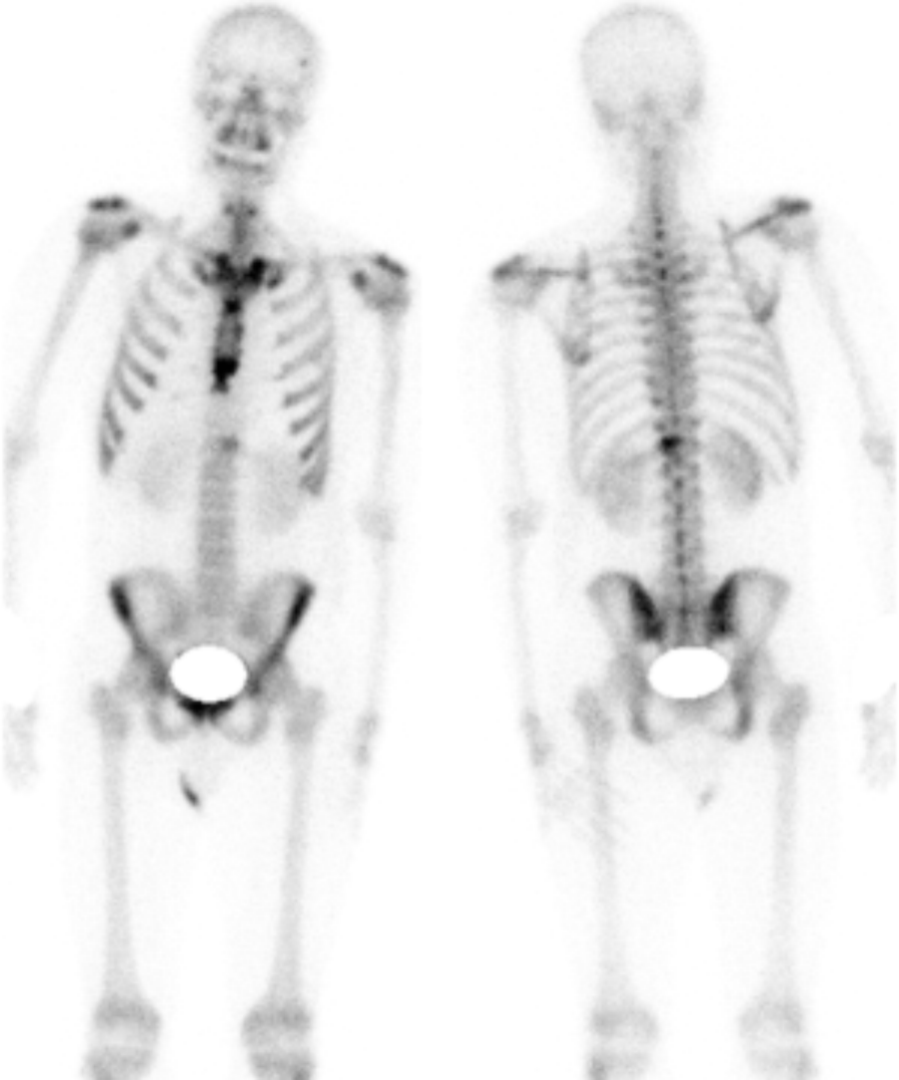
Whole-body bone scan displaying increased uptake of the left tibial/fibular regions, left frontal lobe, and xiphoid process.

A 27-year-old Filipino man presented to Kern Medical with decreased vision in his left eye, low back pain, weakness in his lower extremities, and masses in his paraspinal, supraclavicular, and submandibular regions. Thoracic and abdominal computed tomography scan demonstrated multiple abscesses with involvement of the left supraclavicular lymph nodes and a left paraspinal abscess extending from T7-T12 with penetration and subsequent osteomyelitis of the T12 vertebra (Figures 1 and 2). Whole-body bone scan also showed increased uptake of left fibular and tibial regions, left frontal lobe, and xiphoid process (Figure 3). Incision and drainage of the paraspinal abscess and subsequent staining of the aspirate indicated the presence of double-walled spherules with endosporulation. Coccidioidal serological immunodiffusion showed immunoglobulin M and immunoglobulin G reactivity with a complement fixation titer of ≥1/512. The patient was placed on liposomal amphotericin B for his extraocular disease.

**Figure 4.**
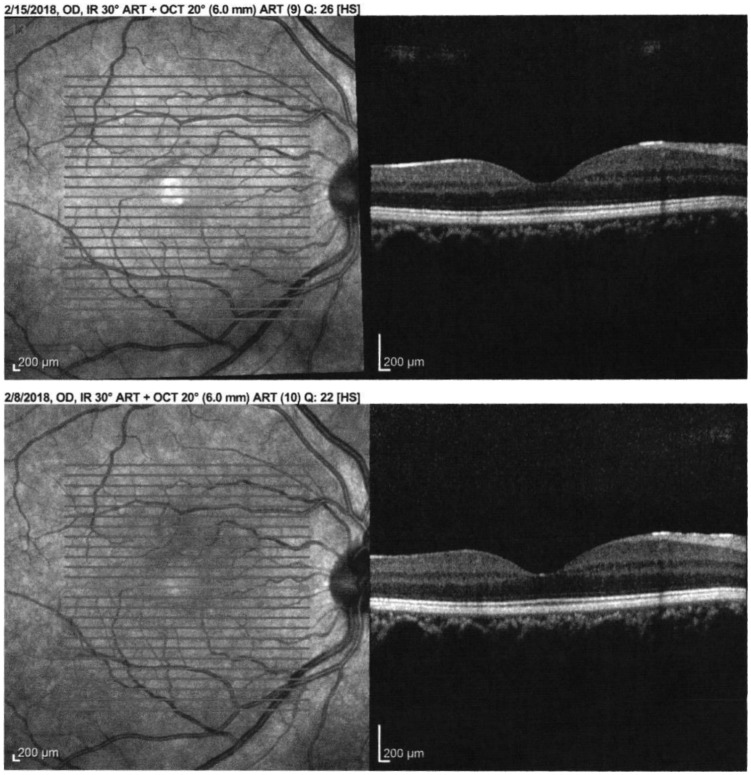
Optical coherence tomography of normal right eye.

**Figure 5.**
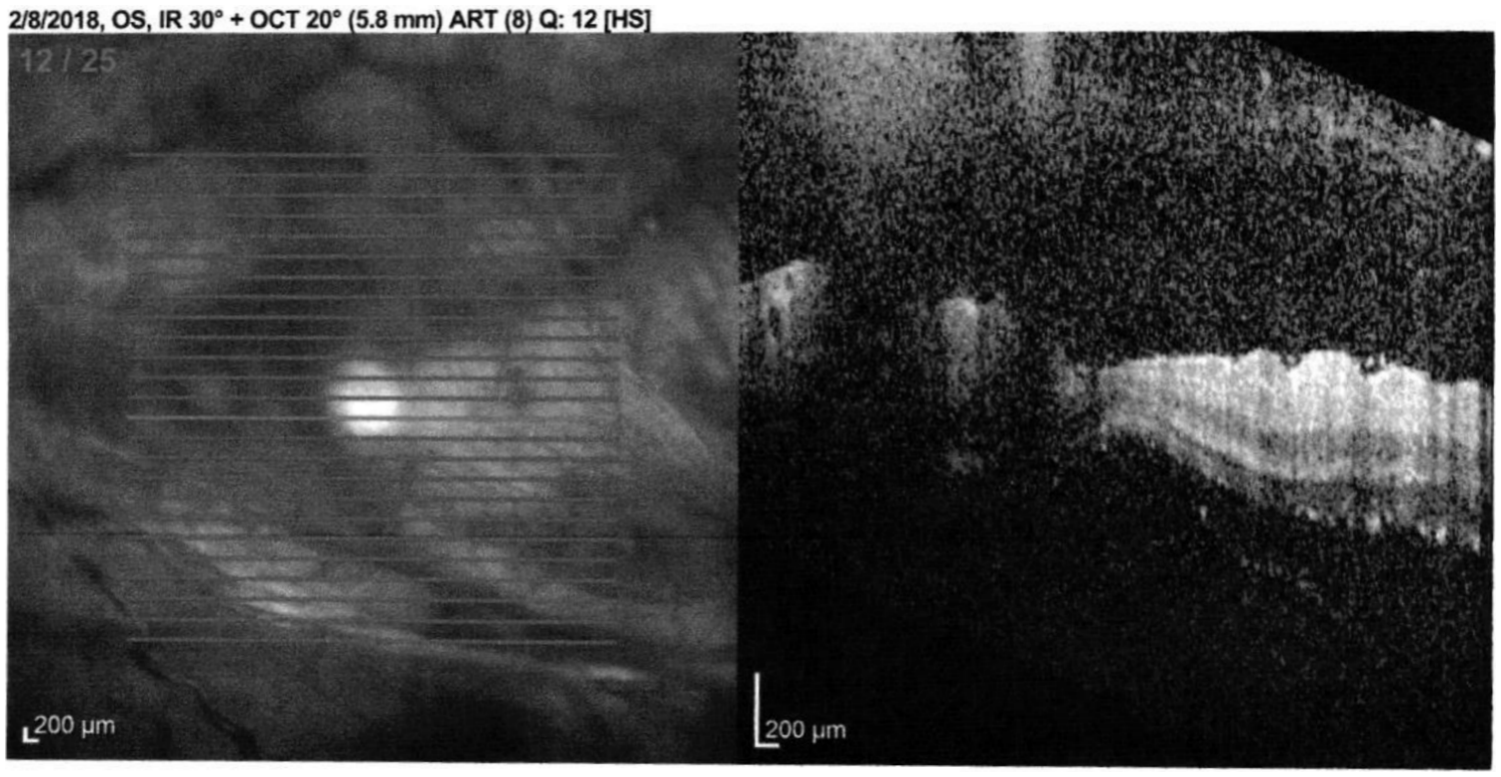
Optical coherence tomography of left eye: multiple vitreous opacities obscuring visualization of the fundal structures.

**Figure 6.**
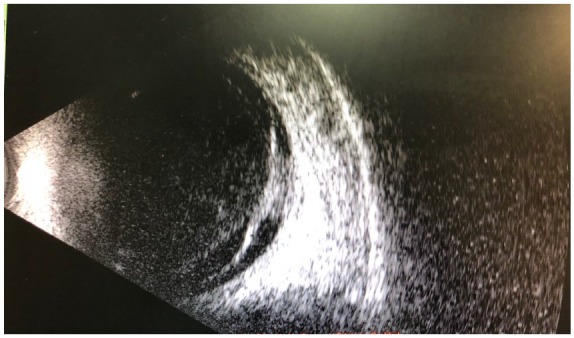
B-scan displaying a tractional/exudative retinal detachment secondary to the coccidioidal choroidal lesion.

The patient started to complain of floaters in his left eye, which progressed until he reported of a total loss of vision in this eye. Ophthalmologic examination discovered “puff balls” in the vitreous overlying the posterior pole in this eye only. The right eye did not have any abnormal findings (Figure 4). He was discharged from the hospital and referred to a retinal specialist as an outpatient. On examination, he had normal visual acuity in the right eye, with light perception vision only in the left eye. Anterior segment examination did not reveal an active anterior uveitis in both eyes. Significant vitreous opacities were found in the left eye (Figure 5). A large, white subretinal lesion was present in the temporal macula. There was significant traction associated with this lesion and a combined tractional/exudative retinal detachment was present, extending inferiorly (Figure 6). Given the history of concurrent coccidioidomycosis infection, the patient was diagnosed with a coccidiomycosis associated chorioretinitis. He was started on intravitreal amphotericin B deoxycholate 5 µg/0.1 mL every 3 days in addition to his systemic treatment.

## Discussion

Unfortunately, our patient has remained poorly adherent to his medical care. He continues to have minimal light perception vision in his left eye and has developed osteomyelitis of his left clavicle. To this date, he has not followed-up with his ophthalmologist and has had no improvement in his vision. The cases reported have had worse outcomes even with proper medical adherence. In 2010, a 64-year-old resident of Southern California with successful treatment of pulmonary coccidioidomycosis developed visual changes 8 years after. Despite aggressive treatment with fluconazole/amphotericin, he eventually lost all vision and the eye was enucleated due to intractable pain. He was found to have a large number of *Coccidioides* sp in the vitreous cavity.^4^ Another case in 1987 of a 12-year-old girl camping near Logan, Arizona, infected with fatal disseminated coccidioidomycosis. On autopsy, she was found to have numerous retinal lesions extending into the vitreous.^6^

Although rarely seen, ocular coccidioidomycosis must be considered in patients with a compatible clinical presentation. Any patient with coccidioidomycosis who has new or unexplained eye symptoms should be immediately referred to an ophthalmologist for examination. Recommended treatment includes vitrectomy and intravitreal amphotericin B deoxycholate. Most patients will be left with extensive choroidal and retinal scarring. Coccidioidomycosis endophthalmitis portends a poor prognosis for vision and often eventuates in enucleation.
